# Factors associated with burnout in military police officers in a city in Paraná

**DOI:** 10.1590/0034-7167-2023-0510

**Published:** 2024-09-06

**Authors:** Beatriz Maria dos Santos Santiago Ribeiro, Luiz Almeida da Silva, Fabio Scorsolini-Comin, Maria Lucia do Carmo Cruz Robazzi, Sérgio Valverde Marques dos Santos, Fábio de Souza Terra, Rita de Cassia de Marchi Barcellos Dalri

**Affiliations:** I Universidade de São Paulo. Ribeirão Preto, São Paulo, Brazil; II Universidade Federal de Catalão. Catalão, Goiás, Brazil; III Universidade do Estado de Minas Gerais. Passos, Minas Gerais, Brazil; IV Universidade Federal de Alfenas. Alfenas, Minas Gerais, Brazil

**Keywords:** Safety, Burnout, Psychological, Police, Occupational Health, Nursing, Seguridad, Agotamiento Psicológico, Policía, Salud Laboral, Enfermería

## Abstract

**Objective:**

to analyze the association between burnout and sociodemographic, work factors, lifestyle habits and health conditions of military police officers in a municipality in the state of Paraná, Brazil.

**Method:**

cross-sectional research with 131 military police officers. Data were analyzed using the Statistical Package for the Social Sciences software and the R program. Chi-square, Fisher’s exact and Poisson Generalized Linear Model tests were used.

**Results:**

most participants (65.6%) had a high level of burnout. In relation to protective factors, those who carried out leisure activities had a 33.6% chance of not developing burnout. Conjugality was also a protective factor. Not practicing physical activity and leisure activities are factors that can contribute to the occurrence of burnout.

**Conclusions:**

important factors and high rates of burnout were observed in the police officers investigated. It is necessary to implement public health policies to reduce burnout with attention focused on this professional category.

## RESUMEN

**Objetivo:**

analizar la asociación entre burnout y factores sociodemográficos, laborales, hábitos de vida y condiciones de salud de policías militares en un município del estado de Paraná, Brasil.

**Método:**

investigación transversal con 131 policías militares. Los datos fueron analizados mediante el software Statistical Package for the Social Sciences y el programa R. Se utilizaron las pruebas de chi-cuadrado, exacta de Fisher y Modelo Lineal Generalizado de Poisson.

**Resultados:**

la mayoría de los participantes (65,6%) presentó un alto nivel de burnout. En relación a los factores protectores, quienes realizaban actividades de ocio tenían un 33,6% de posibilidades de no desarrollar burnout. La conyugalidad era también un factor protector. La no práctica de actividad física y actividades de ocio son factores que pueden contribuir a la aparición del burnout.

**Conclusiones:**

se observaron factores importantes y altos índices de burnout en los policías investigados. Es necesario implementar políticas de salud pública para reducir el burnout con atención centrada en esta categoría profesional.

## INTRODUCTION

Burnout derives from occupational stress. It results from prolonged and chronic stress, and is characterized by three different elements: emotional exhaustion; depersonalization; and lack of professional fulfillment. It can be identified in different workers, especially in people engaged in occupations such as health, education, social services and justice^(1)^.

In this study, the professional category highlighted concerns military police officers (MPOs), who often work at the limit of their resistance capacity, which can cause distress^(2)^. These professionals are subjected to different risks in their daily lives, such as injuries during immobilization of individuals, overcoming obstacles, pursuits, weight of uniforms, weapons, ammunition carriers, ballistic vests and handcuffs, requiring greater physical effort^(3, 4)^. Furthermore, this profession is considered one of the most stressful, given the direct contact with violence, high workload, low wages and inflexible schedules^(4)^.

Studies carried out with Brazilian police officers demonstrated the presence of stress and lack of recognition for the effort made^(5, 6, 7, 8)^. MPOs also have to deal with the lack of structure for carrying out their work, requiring psychological support and guidance that takes their work routines and specificities of the profession as a reference. In certain situations, MPOs may have to intervene in a violent and repressive manner, arousing a feeling of fear rather than respect on the part of the population^(9)^. Other stressors were also observed among MPOs, such as participation in legal proceedings and visits to courts^(10)^.

The Paraná Military Police (MPPR) carries out its activities in defense of life. If necessary, apply immediate criminal offenses, ensure humans’ physical integrity and dignity. Among their functions are developing public safety activities on the streets, working in policing modalities, such as environmental, community, traffic with motorcycles, as well as tactical overt patrols, special operations, radio patrol, school patrol, among other activities^(11)^.

Considering the above and due to the need to seek new knowledge regarding burnout in MPOs, the relevance of analyzing this phenomenon in a specific population, in this case, that of MPPR professionals, is justified. Thus, it is believed that the study can expand scientific knowledge on the subject (MPOs and burnout) as well as allow constructing strategies aimed at promoting health actions in their workplaces and in interaction with other scenarios. For these professionals to be able to work to promote safety, it is necessary that they are, in addition to being trained, motivated, healthy and in full working condition, which involves both physical and mental health preservation. Therefore, this study aimed to answer the following questions: are MPOs affected by burnout in their work environments? What are the sociodemographic and work factors, lifestyle habits and health conditions associated with burnout in PRMP?

## OBJECTIVE

To analyze the association between burnout and sociodemo-graphic and work factors, lifestyle habits and health conditions of MPOs in a municipality in the state of Paraná, Brazil.

## METHOD

### Ethical aspects

In compliance with Resolution 466/12 of the Brazilian National Health Council, this study was approved by the Research Ethics Committee of the first author’s home institution. The Informed Consent Form (ICF) was obtained from all participants before data collection began.

### Study design, period and place

This is a cross-sectional, descriptive-analytical research with a quantitative approach, guided by STrengthening the Reporting of OBservational studies in Epidemiology (STROBE)^(12)^, carried out in a municipality located in northern Paraná^(13)^, which is one of the most populous in the state. It has an area of 199,307.939 km^2^ ^(13)^ and has 28 military battalions, which govern 399 municipalities. Collection was carried out from February to November 2022.

### Population and inclusion criteria

The study population was made up of MPPR members working in a battalion belonging to a municipality in northern Paraná, with a total of 215 MPOs. All 215 MPOs were invited to participate in the study. To participate, they had to be a MPO in the state network in northern Paraná and have at least one year’s experience in this occupation. Those who were on sick leave, absent during the period of data collection and with less than a year in this occupation were excluded. Based on this recruitment and the application of the inclusion and exclusion criteria by the researchers, 131 MPOs participated in the present study, composing a convenience sample.

### Study protocol

The following stages were followed in this study: 1) recruitment of MPOs by face-to-face and/or telephone means; 2) checking the number of participants; 3) completion, by participants, of instruments relating to sociodemographic, work data, lifestyle habits and health conditions and the Oldenburg Burnout Inventory (OLBI). This was completed using Google Forms®, using a link shared by the research team with the authorization of immediate superiors. From this link, participants had access to the ICF and, if they agreed with the terms of this study, also to the data collection instruments. An instrument was applied to identify sociodemographic, work data, lifestyle habits and health conditions, developed by the authors, containing the variables sex, age, religious belief, skin color, number of children, marital status, education, family and individual income, professional practice time, length of experience in the area of public security, weekly working hours, number of employment relationships, work shift, absence in the last semester for health reasons, last vacation, physical activities, leisure, smoking, alcoholism, chronic disease, use of medication and frequent health problems. The authors relied on an article published in 2022^(14)^ that verified the association between burnout syndrome and occupational violence in teachers in the same region as this study to prepare the instrument.

To identify the presence and indices of burnout, we chose an instrument validated for Brazil and with good reliability indices^(15)^, the OLBI, which assesses the characteristics that can trigger burnout and are associated with relationships and working conditions through a scale that contains 13 questions and two factors, such as exhaustion, with six questions, and disengagement from work, with seven. It can be applied in any occupational context. This scale was originally developed in 1999 in German, being translated and validated in Brazil in 2018. The license to use the OLBI in this study was requested and granted by the authors who validated it in Brazil.

### Statistical analysis

A double-entry database was created, and the analyzes were carried out using the Statistical Package for the Social Sciences (SPSS) version 25 and the R program version 3.4.0. Data were analyzed using descriptive statistics, calculating frequency and percentage for categorical variables. They were categorized dichotomously, taking into account the mean parameter for class configuration. In order to analyze OLBI reliability for the present sample, Cronbach’s alpha analysis was carried out, which revealed an adequate index (α=0.925).

Subsequently, analysis was carried out using Fisher’s exact test to verify the existence of an association between burnout dimensions and personal and occupational characterization, lifestyle habits and health conditions. The significance level adopted for these analyzes was α ≤ 0.05.

Aiming to standardize the variable characterized as outcome, taking into account the distribution of positive occurrences (having burnout), established in four domains, it was decided to carry out the categorization between not having burnout (without burnout, with disengagement, with exhaustion) and having burnout (with burnout). Using chi-square and Fisher’s exact tests, an analysis of the Odds Ratio and possible associations between variables, personal and occupational characteristics, lifestyle habits and health conditions with burnout syndrome was carried out.

In order to estimate a prediction model for belonging to the category of people who had burnout, the Poisson Generalized Linear Model with robust variance was used, thus obtaining the prevalence ratio. All data were considered significant with a p-value ≤ 0.05.

## RESULTS

A total of 131 police officers participated in this study, 87.0% (n=114) male, 85.4% (n=112) aged between 31 and 59 years old, 64.1% (n=84) white, 62, 5% (n=82) Catholic, 63.3% (n=83) with one to two children and 81.6% (n=107) married or with partners. As for training, 45.0% (n=59) had a graduate degree. Regarding monthly family income, 47.3% (n=62) reported receiving more than five minimum wages per month (US$ 1,212.00).

Using alcoholic beverages was found in 53.4% (n=70); no participant reported using illicit drugs; and only 8.4% (n=11) were smokers. In relation to the health-illness-care processes, 93.8% (n=123) did not have a chronic illness; 73.2% (n=96) did not use continuous medications; 87.0% (n=114) stated that they did not have frequent health problems; and 76.3% (n=100) practiced leisure activities. Regarding mental health, 65.6% (n=86) showed higher rates of burnout, while the remainder focused on other responses (without burnout, with detachment and with exhaustion). Only the leisure activity variable was associated with burnout (p = 0.009).

A little more than half of the police officers had more than 10 years of experience in the MPPR (51.9%, n=68), and 57.2% (n=75) had more than 10 years of experience in the area of public security (Table 1). The majority, i.e., 79.3% (n=104), worked more than 40 hours a week; 76.3% (n=100) had no other employment relationship; 90.0% (n=118) worked rotating shifts; 54.9% (n=72) did not need to be absent from work in the period of one year before data collection; and the same number practiced physical activity more than three times a week. At the time of data collection, 73.2% (n=96) had already taken vacation that year.

**Table 1:** Distribution of the occurrence of burnout according to sociodemographic, work variables, lifestyle habits and health conditions. Paraná, Brazil, 2022 (N= 131)

Burnout		Burnout					
		Yes		No		OR(CI)	*p* value
		f	%	f	%		
Sex	Male	86	86.9	28	87.5	1.044 (0.418-2.607)	0.926
	Female	13	13.1	4	12.5		
Skin color	White	64	64.6	20	62.5	1.023(0.833-1.257)	0.826
	Non-white	35	35.4	12	37.5		
Religious belief	With belief	95	96.0	30	93.8	1.140(0.642-0.025)	0.603
	Without belief	4	4.0	2	6.3		
Marital status	With partner	79	79.8	28	87.5	1.570(0.717-1.095)	0.328
	Without partner	20	20.2	4	12.5		
Education	Higher education	80	80.8	24	75.0	1.093(0.837-1.427)	0.480
	High school	19	19.2	8	25.0		
Family income	Up to 5 MW	52	52.5	17	54.8	1.073(0.579-1.991)	0.822
	>5 MW	47	47.5	14	45.2		
Individual income	< 3 MW	9	9.1	2	6.3		
	3 or more MW	90	90.9	30	93.8	1.091(0.811-1.468)	0.614
Years of professional practice	6 -10	50	50.5	13	40.6		
	>10	49	49.5	19	59.4	1.101(0.907-1.338)	0.331
Years of experience in public safety	Up to 10	45	45.5	11	34.4		
	>10	54	54.5	21	65.6	1.116(0.922-1.352)	0.271
Has another job	No	72	72.7	28	87.5	2.170(0.825-5.708)	0.087
	Yes	27	27.3	4	12.5		
Work shift	Rotating	88	88.9	30	93.8	1.653(0.445-6.135)	0.424
	Fixed	11	11.1	2	6.3		
Days absent from work	No	53	53.5	19	59.4	1.198(0.647-2.217)	0.564
	Yes	46	46.5	13	40.6		
Alcoholic beverages	No	44	44.4	17	53.1		
	Yes	55	55.6	15	46.9	1.301(0.711-2.378)	0.392
Smoking	No	91	91.9	29	90.6	1.043(0.716-1.518)	0.818
	Yes	8	8.1	3	9.4		
Chronic disease	No	92	92.9	31	96.9	2.016(0.314-12.932)	0.418
	Yes	7	7.1	1	3.1		
Continuous use of medications	No	71	71.7	25	78.1	1.302(0.619-2.738)	0.476
	Yes	28	28.3	7	21.9		
Frequent health problem	No	84	84.8	30	93.8	2.237(0.587-8.522)	0.193
	Yes	15	15.2	2	6.3		

*Fisher’s exact test; minimum wage (MW) on the date of data collection: US$253.56 (US dollar exchange rate on 06/02/2022).

It was found that there was no association between occupational variables and burnout dimensions. In Table 1, MPOs who did not have another job were approximately twice as likely to not experience burnout than those who did. Not having a chronic disease was approximately twice as likely to not develop burnout, and those who did not have frequent health problems were approximately twice as likely to not experience burnout. The leisure activity variable showed an association, indicating that those who practiced leisure activities had a 33.6% chance of not developing burnout when compared to those who did not practice it (OR: 1.336, p = 1.008). These data are summarized in Table 1.

The variables sex and age were kept in the model, as they were considered confounding, however they did not contribute to its construction. The variables days absent, vacations that year, having a chronic disease and age showed a marginal or borderline association. The rotating shift variable proved to be a protective factor for the occurrence of burnout, as it presented a negative coefficient (β= −0.363, p = 0.019) (Table 2).

**Table 2:** Analysis of individual factors in predicting the burnout outcome variable (yes or no). Paraná, Brazil, 2022 (N= 131)

Parameter	β	*p* value	PR	(95%) CI	
				Lower	Upper
(Intercept)	1.372	<0.001	3.944	2.162	7.194
Sex					
Male	0.024	0.755	1.024	0.883	1.187
Female	Ref.				
Work shift					
Rotating	−0.363	0.019	0.695	0.513	0.943
Fixed	Ref.				
Days absent					
< 15 days	−0.101	0.083	0.904	0.807	1.013
>15days	Ref.				
Holiday this year					
No	0.078	0.083	1.082	0.990	1.182
Yes	Ref.				
Physical activity					
No	0.100	0.044	1.106	1.003	1.219
Yes	Ref.				
Has a chronic disease					
No	−0.166	0.058	0.847	0.713	1.006
Yes	Ref.				
Age (years)	−0.008	0.066	0.992	0.983	1.001
Performs leisure activities					
No	0.077	0.049	1.080	1.000	1.165
Yes	Ref.				
Marital status					
With partner	-0.121	0.025	0.886	0.797	0.985
Without partner	Ref.				

*Poisson regression with robust variance; Ref. – reference; β=regression coefficient; PR – prevalence ratio; CI – Confidence Interval.

Not practicing physical exercise contributed positively to the occurrence of burnout (β= −0.100, p = 0.044), just as not performing leisure activities also contributed positively to its occurrence (β= 0.077, p = 0.049). Marital status with a partner was protective against developing burnout (β= −0.121, p = 0.025) (Table 2).

Table 2, below, presents regression analysis. Variables that demonstrated vulnerability to the development of burnout can be noted in the model, such as fixed work shifts, having a chronic disease, not carrying out leisure activities or practicing physical activities and not having a partner.

## DISCUSSION

Most participants in this study (87.0%) demonstrated higher rates of burnout, a finding that is reported in the scientific literature^(16)^, especially considering the various markers associated with the daily life of this profession, such as the issue of secrecy and performance faced with stressors that are not always controllable.

Women who work as MPOs tend to have higher levels of stress when compared to men in this profession^(17)^, which may be due to the other social roles occupied according to gender in our country. It is noteworthy that, in the present study, there was a greater number of male participants (85.4%), which was already expected, as the military professional category is mainly made up of men. A study carried out in 2019 in the state of Rio Grande do Sul also observed this prevalence^(18)^.

Although scientific literature may suggest associations between age, skin color and burnout^(19)^, the present study showed only a marginal/borderline association between these variables. Regarding religiosity/spirituality, the majority of the sample identified themselves as Catholic (62.5%). Religiosity/spirituality can be understood as a resource used at the individual and collective levels, which seeks to face adverse life events, which can have emotional and social repercussions. Thus, it is believed that religious belief can be a resource for participants who have some belief, as religiosity can give new meaning to the meaning of human existence and expand concepts and awareness. MPO’s search through religiosity and the development of spirituality can help in the space for construction and reflection regarding each person’s current and future life moment^(20)^.

It was found that 63.3% of MPOs had one to two children, data close to the results presented by a study that aimed to assess the perception of physical activity indices and burnout indicators in MPOs in the city of Belo Horizonte in the state of Minas Gerais (MG), with 61.0% of participants having children™. Performing physical activities is a protective factor against burnout, a finding emphasized in the present study. In relation to parenting, there is a consensus in the literature that being a father or mother is a possible cause of protection or a lower rate of diseases such as burnout^(22)^. However, this association was not significant in the present study. It is suggested here that a protective relationship can occur when parenting is positive and when relationships between parents and children are healthy, with a lower rate of conflict. To support this hypothesis, the parenting variable should have been explored more consistently in the present investigation.

In relation to the level of education, although, to be admitted to Military Police, only completion of high school is required, it was found, in the present investigation, that some of the police officers had improved, i.e., 45% of them had a graduate degree. It should be noted that there is an offer of in-person and distance undergraduate/graduate opportunities in several higher education institutions in the city where the study was carried out, as well as ease of movement between work and educational institutions in the city where the police officers reside, discount on monthly fees for being a MPO and scholarship offers for this population. This scenario may have contributed to the increase in the level of education in this sample. However, a study carried out in Belo Horizonte, MG, with 195 MPOs, showed that the majority of them only had high school^(21)^. Research carried out with teachers with burnout demonstrated that the higher the level of education, the greater the likelihood of developing it^(14)^.

In relation to monthly individual and family income, in this research, it was found that the investigated population received above three minimum wages, based on the value of US$253.56 (year 2022), i.e., an amount lower than the Brazilian middle class^(23)^. Research carried out in Rio Grande do Sul showed that there was job dissatisfaction among MPOs in relation to wage and career promotions^(24)^. Wage range can influence the risk of developing burnout. Lower wages can lead to experiencing the tension triggered by an income that is insufficient for their needs. Higher income is probably the result of more than one employment relationship, a fact that can lead to physical and/or mental exhaustion^(25)^, due to accumulation of functions and more rigid working hours.

Unlike the study carried out in the city of Belo Horizonte, MG, whose MPOs had low adherence to physical activity^(21)^, the results of this study showed that the majority practiced physical activity more than three times a week. In agreement with these results, not practicing physical exercise can contribute positively to the occurrence of burnout; therefore, the higher the level of physical activity, the lower the chances of developing burnout^(26)^.

The majority of participants drank alcohol (53.4%); none reported using illicit drugs; and the minority were smokers. Substance abuse represented by alcohol dependence is commonly associated with depressive disorders and burnout^(27)^. Policing activities can cause sleep disorders and, consequently, administrative errors, dangerous driving of vehicles, uncontrolled feelings of anger, security breaches due to absenteeism and fatigue and constant exposure to human tragedy, leading to the risk of MPOs developing mental disorders, with the potential for alcohol and other drug abuse^(28)^.

The reality of this activity, countless times, is permeated by tension, pressure, demands, psychological distress, dissatisfaction with working conditions, lack of preparation for the role and “unconditional” obligation not to demonstrate fragility, These factors are related to the emergence of psychological disorders^(29)^, which can also increase the chances of professionals developing symptoms of burnout, as found in this study.

In this research, 93.8% of police officers did not have a chronic disease, a protective factor for burnout, and 73.2% did not use continuous medications. Furthermore, 87.0% stated that they did not have frequent health problems; however, a large number of MPOs with burnout were observed. It was observed that not having a chronic disease makes a person approximately twice as likely to not develop burnout. Studies have shown that the factors related to the disease of MPOs were not being able to provide help in a timely manner, being powerless in the face of deaths of people who were unable to help, negative interpretations conveyed to the MPO, lack of leisure, low quality of life and victimization. Such situations can interfere with the quality of life of these workers, causing health problems and use of medications^(30, 31, 32, 33)^.

MPO’s work can cause them to be separated from society, family, friends and interpersonal relationships^(34)^ and, often, the conditions in which they work are precarious^(4)^. The fact that they do not carry out leisure activities significantly contributes to the occurrence of burnout, and it was observed, in this study, that leisure activities lead to 33.6% more chances of not developing burnout when compared to those who did not practice such activities.

Research that aimed to investigate the rates of occupational stress and work engagement in MPOs in Paraná also revealed that the majority of participants were married or had a partner^(4)^. In relation to marital status, some research attests that marital status is a condition that implies less disposition to burnout symptoms, a fact supported by the present study^(22)^. Thus, having a partner was a protective factor against the occurrence of burnout, which can be explained both by the fact that this partner offers emotional support and by the fact that these interpersonal relationships offer satisfaction and a sense of well-being. In a profession strongly marked by stress and the need for confidentiality, sharing everyday life with a partner can be a factor that predisposes professionals to greater openness and greater care in relation to their mental health.

This study also showed that, in MPOs that had more than 10 years of experience and more than 10 years of experience in the area of public security, with a weekly workload of more than 40 hours and with significant experience and time working, burnout was present. Research carried out in the United States demonstrated that the longer the service, the greater the stress in the work environment^(16)^, in addition to a greater feeling of anxiety and distress^(35)^.

A review study showed that more experienced workers feel safe, have control over their work environment and deal better with work demands, being more protected against burnout^(36)^. However, in the present research, the findings disagreed with this result reported in the scientific literature, given that there was no association between age and length of experience with the burnout variable.

It is worth mentioning that MPOs who did not have another job were approximately twice as likely to not experience burnout than those who did. Therefore, having a work-only relationship proved to be a protective factor for burnout^(22)^. It is noteworthy that 76.3% of those surveyed had only one job. However, there are mentions in the literature that low wages make MPOs seek to increase their income with other work^(37)^, which can favor the presence of burnout.

In this study, 90.0% of MPOs worked rotating shifts, and this factor was protective against the occurrence of burnout. Factors such as lack of days off, working in shifts, changing shifts without prior notice, dealing with rigid hierarchy and bureaucracy, caring for abused children, having to take someone’s life if necessary, seeing co-workers killed while on duty, going to court on their days off, lack of supervisor support, inefficient staffing, working on weekends and work demands are related to the emergence of psychological symptoms^(30, 31, 32)^.

In this research, 54.9% of those surveyed were not absent from work in the year of data collection, and 73.2% had already taken vacation that same year. Such variables and their prevalence ratio in the population showed a marginal/borderline association with burnout. Adjusting days off, not letting vacations accumulate, adjusting wages and providing a housing and health plan for MPOs can be actions that avoid harm to these professionals’ health^(38)^.

Given the results presented so far, it is possible to infer that police activities require attention from both managers and public policies, with the objective of managing stressful factors and consequently controlling burnout in this population. MPOs’ work is of great importance for the country, and its nature goes beyond physical conditioning, requiring good emotional performance^(17)^.

In this context, it is important to mention that public policies at federal, state and municipal levels to combat this phenomenon are essential. It is suggested to use strategies that promote reduced work overload, good interpersonal relationships between hierarchical levels and improved working conditions and work organization to reduce factors associated with burnout. Above all, it highlights the importance of paying close attention to the occupational risks to which these workers are exposed, investing and making resources available so that there are more healthcare professionals in an interdisciplinary way, contributing to health promotion of these workers responsible for public safety.

### Study limitations

As limitations of this study, it is reported that this research has a cross-sectional design and does not allow establishing relationships between cause and effect. It is also important to highlight the difficulty of finding investigations on the topic in question, which can limit the discussion of findings and their comparison in the scientific literature.

### Contributions to health, nursing, or public policy

It is assumed that the present study can support the understanding of the factors that lead MPOs to experience burnout. It presents itself as a possibility for new studies, mainly in the areas of worker health, mental health and public safety, by encouraging a rethinking of MPOs’ health with a comprehensive view. These data can, together, promote advances in the production of scientific and practical knowledge regarding this topic, enabling the development of measures and policies aimed at this population. There is a need for projects that aim to increase the quality of work life of these professionals and incentives for scientific research, in order to assess results and scope of their practice in different regions.

It greatly contributes to ensuring that these professionals are not silenced in relation to their psychological distress, which is one of the commitments reinforced by this research and the data shared here. Strategies and public policies aimed at preventing burnout are urgent as well as interventionist actions that reduce burnout and aim to maintain MPOs’ physical and mental health as: work environment monitoring; offering integrative and complementary practices; monitoring of these police officers by healthcare professionals in an interdisciplinary manner; promotion of support networks; and discussion of this topic with managers and society in general, emphasizing the need for these professionals to be heard and cared for.

## CONCLUSIONS

In this study, high rates of burnout were observed in the police officers investigated. Important results were highlighted, such as that not having another job and not having a chronic disease leads to a greater chance of not developing burnout as well as that those who did not have frequent health problems.

The rotating shift variable proved to be a protective factor against the occurrence of burnout, as well as marital status (having a partner), reinforcing the importance of both work factors (shift) and satisfactory interpersonal relationships (conjugality) for maintaining emotional health and quality of life, which may be reflected in lower burnout rates when these protective factors are present.

Similarly, but in the opposite direction, not practicing physical exercise and not carrying out leisure activities contributes to the occurrence of burnout. In view of these findings, it is essential that discussions about mental health occur in these professionals’ work, integrating both work elements that can be adjusted, such as work shifts, and encouraging investment in actions that go beyond the work context, such as encouraging physical exercise, leisure activities and strengthening interpersonal relationships, among which we place, from this study, conjugality.

## References

[1] Maslach C, Leiter MP (2016). stress: concepts, cognition, emotion, and behavior.

[2] Pessoa DR, Dionísio AG, Lima LDV, Soares RMN, Silva JM (2016). Incidência de distúrbios musculoesqueléticos em policiais militares pelo impacto do uso de colete balístico. Rev Univap.

[3] Guedes IFI (2017). Reconvocação de Policiais Militares para o Serviço Administrativo na PMSGO: estudo dos Colégios Hugo C. Ramos e Vasco dos Reis. Rebesp [Internet].

[4] Pelegrini A, Cardoso TE, Claumann GS, Pinto AA, Felden EPG (2018). Perception of work conditions and occupational stress among civil and military police officers ofspecial operations units. Cad Bras Ter Ocup.

[5] Souza KMO, Azevedo CS, Oliveira SS (2017). A dinâmica do reconhecimento: estratégias dos Bombeiros Militares do Estado Rio de Janeiro. Saude Deb.

[6] Machado CE, Traesel ES, Merlo ÁRC (2017). Profissionais da Brigada Militar: vivências do cotidiano e subjetividade. Psicol Arg[Internet].

[7] Lima AS, Farah BF, Teixeira MTB (2018). Análise da prevalência da Síndrome de Burnout em profissionais da atenção primária em saúde. Trab Educ Saúde.

[8] Mota BC, Campos BL, Souza EL, Peixoto RF, Braga V (2019). Violência e mortes de policiais. J Eletron Fac Int Viana Jr.

[9] Durão S (2016). Esquadra de polícia.

[10] Pawlowytsch PWM, Batista LR, Batista FCN (2013). Um estudo exploratório sobre o Estresse nos Policiais Militares de uma Cidade Catarinense. Saude Meio Amb: Rev Interd.

[11] Klemps F (2021). Policial militar X agente de autoridade de trânsito. BJD.

[12] Rede Equator (2007). Checklist STROBE para estudos transversais.

[13] Instituto Brasileiro de Geografia e Estatística (IBGE) (2017). Cidades e Estados.

[14] Ribeiro BM, Martins JT, Moreira AA, Galdino MJ, Lourenço MC, Dalri RC (2022). Association between burnout syndrome and workplace violence in teachers. Acta Paul Enferm.

[15] Schuster MS, Dias VV (2018). Oldenburg Burnout Inventory-validação de uma nova forma de mensurar Burnout no Brasil. Cien Saude Colet.

[16] Rose T, Unnithan P (2015). In or out of the group? police subculture and occupational stress. Policing: An J Police.

[17] Rodrigues APG, Pinheiro DM, Duarte LR (2021). A vulnerabilidade ao estresse apresentada pelo policial militar diante do clima organizacional da Corporação. Rebesp.

[18] Hoch SS (2019). “Belas, empoderadas e armadas”: o preconceito patriarcal contra a profissão de Policial Militar Feminina. Coisas do Gênero: Rev Est Femin Teol Relig[Internet].

[19] Talarico BVH, Silva BI, Leonel HPH, Carvalho NHR, Pavão PK (2021). Fatores associados ao bem-estar em profissionais da atenção primária. Rev Bras Promoc Saúde.

[20] Ribeiro BMSS, Scorsolini-Comin F, Souza SR (2021). Síndrome de burnout em profissionais da enfermagem de unidade de terapia intensiva na pandemia da COVID 19. Rev Bras Med Trab.

[21] Soares DS, Melo CC, Soares JLSS, Noce F (2019). Influência da atividade física no burnout em policiais militares. J Phys Educ.

[22] Silva FG, Silva VA, Martins JT, Santana MAS, Ribeiro BMSS (2020). Burnout syndrome in nursing professionals in a neonatal intensive therapy unit. Rev Enferm UFPI.

[23] Instituto Brasileiro de Geografia e Estatística (IBGE) (2021). Em 2021, rendimento domiciliar per capita cai ao menor nível desde 2012[Internet].

[24] Almeida DM, Lopes LFD, Costa VMF, Santos RCT (2016). Job Satisfaction of the Rio Grande do Sul Military Policemen: a Quantitative Study. Psicol Cienc Prof.

[25] Luz LM, Torres RRB, Sarmento KMVQ, Sales JMR, Farias KN, Marques MB (2017). Síndrome de Burnout em profissionais de serviço de atendimento móvel de urgência. Rev Pesqui Cuid Fundam.

[26] Farias GO, Mota ID, Marinho APR, Both J, Veiga MB (2019). Relação entre atividade física e Síndrome de Burnout em estudantes universitários: revisão sistemática. Pensar a prática.

[27] González-Urbieta I, Alfonzo A, Aranda J, Cameron S, Chávez D, Duré N (2020). Síndrome de Burnout y dependencia al alcohol en estudiantes de Medicina. Med Clín Soc.

[28] Fontana RT, Mattos GD (2016). Vivendo entre a segurança e o risco: implicações à saúde do policial militar. Cienc Cuid Saúde.

[29] Gomes AR, Afonso JMP (2016). Occupational stress and Coping among Portuguese Military Police Officers. APL.

[30] Andrew ME, Violanti JM, Gu JK, Fekedulegn D, Li S, Hartley TA (2017). Policework stress ors and cardiac vagal control. Am J Hum Biol.

[31] Fekedulegn D, Burchfiel CM (2016). Correlates of hopelessness in the high suicide risk police occupation. Police Pract Res.

[32] Lipp MEN, Costa KRSN, Nunes VO (2017). Estresse, qualidade de vida e estress ores ocupacionais de policiais: síntomas mais frequentes. Rev Psicol Organ Trab.

[33] Shiozaki M, Miyai N, Morioka I, Utsumi M, Hattori S, Koike H (2017). Job stress and behavioral characteristics in relation to coronary heart disease risk among Japanese police officers. Industrial Health.

[34] Miranda D, Guimarães TO (2016). Suicídio policial: o que sabemos?. DILEMAS [Internet].

[35] Santos FBD, Lourenção LG, Vieira E, Ximenes FRG, Oliveira AMND, Oliveira JFD (2021). Estresse ocupacional e engajamento no trabalho entre policiais militares. Ciênc Saúde Coletiva.

[36] Bakker AB, Demerouti E, Sanz-Vergel AI (2014). Burnout and Work Engagement The JD-R Approach. Annu Rev Organ Psychol Organ Behav.

[37] Marinho MT, Souza MBCA, Santos MMA, Cruz MAA, Barroso BIL (2018). Fatores geradores de estresse em policiais militares: revisão sistemática. Rev Fam Cic Vid Sau Com Soc.

[38] Minayo MCS, Souza RE, Constantino P (2008). Missão prevenir e proteger, condições de vida, trabalho e saúde dos policiais militares do Rio de Janeiro.

