# Supplementary material for: Factors associated with burnout in military police officers in a city in Paraná
Source: Rev Bras Enferm. 2024 Sep 6;77(4):e20230510. doi: 10.1590/0034-7167-2023-0510 (PMC11382680; doi:10.1590/0034-7167-2023-0510)
Supplement: Translation

# Translation: Fatores associados ao burnout em policiais militares de uma cidade do Paraná

**DOI:** 10.1590/0034-7167-2023-0510pt

**Authors:** Beatriz Maria dos Santos Santiago Ribeiro, Luiz Almeida da Silva, Fabio Scorsolini-Comin, Maria Lucia do Carmo Cruz Robazzi, Sérgio Valverde Marques dos Santos, Fábio de Souza Terra, Rita de Cassia de Marchi Barcellos Dalri

**Affiliations:** I Universidade de São Paulo. Ribeirão Preto, São Paulo, Brasil; II Universidade Federal de Catalão. Catalão, Goiás, Brasil; III Universidade do Estado de Minas Gerais. Passos, Minas Gerais, Brasil; IV Universidade Federal de Alfenas. Alfenas, Minas Gerais, Brasil

**Keywords:** Segurança Pública, Esgotamento Psicológico, Policiais, Saúde Ocupacional, Enfermagem

## Abstract

**Objetivo:**

analisar a associação entre *burnout* e fatores sociodemográficos, laborais, hábitos de vida e condições de saúde de policiais militares de um município do estado do Paraná, Brasil.

**Método:**

pesquisa transversal com 131 policiais militares. Os dados foram analisados por meio do *software Statistical Package for the Social Sciences* e do programa R. Utilizaram-se os testes qui-quadrado, exato de Fisher e Modelo Linear Generalizado de Poisson.

**Resultados:**

a maioria dos participantes (65,6%) apresentou elevado índice de *burnout.* Em relação aos fatores protetivos, quem realizava atividades de lazer possuía 33,6% chances de não desenvolver *burnout.* A conjugalidade também foi um fator protetivo. Não praticar atividade física e atividades de lazer são fatores que podem contribuir para ocorrência de *burnout.*

**Conclusões:**

observaram-se fatores importantes e índices elevados de *burnout* nos policiais investigados. Faz-se necessária a implementação de políticas públicas de saúde para redução do *burnout* com atenção voltada a essa categoria profissional.

## INTRODUÇÃO

O *burnout* deriva do estresse ocupacional. É resultante de estresse prolongado e crônico, sendo caracterizado por três diferentes elementos: exaustão emocional; despersonalização; e falta de realização profissional. Pode ser identificado em diferentes trabalhadores, sobretudo em pessoas engajadas em ocupações como as da área saúde, da educação, do serviço social e da justiça^(1)^.

Neste estudo, a categoria profissional destacada é a dos policiais militares (PMs), que trabalham, muitas vezes, no limite de sua capacidade de resistência, o que pode gerar sofrimento^(2)^. Esses profissionais estão submetidos a diferentes riscos em seu cotidiano, tais como lesões durante a imobilização dos indivíduos, ultrapassagem de obstáculos, perseguições, peso do fardamento, armamento, carregadores de munição, colete balístico e algemas, havendo necessidade de maior esforço físico^(3, 4)^. Além disso, essa profissão é considerada uma das mais estressantes, haja vista o contato direto com a violência, elevada carga horária de trabalho, baixos salários e inflexibilidade de escalas^(4)^.

Estudos realizados com policiais brasileiros demonstraram a presença de estresse e falta de reconhecimento pelo esforço realizado^(5, 6, 7, 8)^. Os PMs também têm que lidar com a falta de estrutura para o desenvolvimento do trabalho, necessitando de apoio psicológico e de orientações que tomem como referência suas rotinas de trabalho e especificidades da profissão. Em determinadas situações, os PMs podem ter que intervir de modo violento e repressivo, despertando um sentimento de medo e não de respeito por parte da população^(9)^. Também foram observados outros fatores estressantes entre os PMs, tais como a participação em processos judiciais e as visitas aos tribunais^(10)^.

A Polícia Militar do Paraná (PMPR) desempenha sua atuação em defesa da vida. Se necessário, aplica infrações penais imediatas, zela pela integridade física e a dignidade da pessoa humana. Entre suas funções, estão desenvolver atividades de segurança pública nas ruas, atuando nas modalidades de policiamento, como o ambiental, comunitário, de trânsito com motocicletas, além de rondas ostensivas táticas, operações especiais,radiopatrulha, patrulha escolar, bem como outras atividades^(11)^.

Diante do exposto e devido à necessidade de buscar novos conhecimentos relativos ao *burnout* em PMs, justifica-se a pertinência de analisar esse fenômeno em uma população específica, no caso, a dos profissionais da PMPR. Assim, acredita-se que o estudo possa ampliar o conhecimento científico sobre a temática (PMs e *burnout*), bem como permitir a construção de estratégias que visem à promoção de ações de saúde em seus locais de trabalho e na interação com outros cenários. Para que esses profissionais possam atuar na promoção da segurança, é mister que estejam, além de capacitados, motivados, saudáveis e em plenas condições para o trabalho, o que envolve tanto a preservação da saúde física quanto a da mental. Assim sendo, pretendeu-se, com este estudo, responder às seguintes questões: os PMs são acometidos pelo *burnout* em seus ambientes de trabalho? Quais os fatores sociodemográficos e laborais, hábitos de vida e condições de saúde associados ao *burnout* na PMPR?

## OBJETIVO

Analisar a associação entre o *burnout* e os fatores sociodemográficos e laborais, hábitos de vida e condições de saúde de PMs de um município do estado do Paraná, Brasil.

## MÉTODO

### Aspectos éticos

Em cumprimento à Resolução nº 466/12 do Conselho Nacional de Saúde, este estudo foi aprovado por Comitê de Ética em Pesquisa da instituição de origem da primeira autora. O Termo de Consentimento Livre e Esclarecido (TCLE) foi obtido de todos os participantes antes do início da coleta de dados.

### Desenho, período e local do estudo

Pesquisa transversal, descritivo-analítica e de abordagem quantitativa, norteada pelo protocolo *STrengthening the Reporting ofOBservationalstudies in Epidemiology* (STROBE)^(12)^, realizada em um município situado no norte do Paraná^(13)^, que é um dos mais populosos do estado. Possui área de 199.307,939 km^2^ ^(13)^ e conta com 28 batalhões militares, que regem 399 municípios. A coleta foi realizada de fevereiro a novembro de 2022.

### População e critérios de inclusão

A população do estudo foi composta por integrantes da PMPR atuantes em um batalhão pertencente a um município do norte do Paraná, com um total de 215 PMs. Todos os 215 PMs foram convidados a participar do estudo. Para participar, era preciso ser PM da rede estadual do norte do Paraná e ter experiência de, pelo menos, um ano nessa ocupação. Foram excluídos os que se encontravam em licença saúde, afastamentos no período da coleta de dados e com menos de um ano nessa ocupação. A partir desse recrutamento e da aplicação dos critérios de inclusão e exclusão pelos pesquisadores, 131 PMs participaram do presente estudo, compondo uma amostra de conveniência.

### Protocolo do estudo

As seguintes etapas foram seguidas neste estudo: 1) recrutamento dos PMs por meio presencial e/ou telefônico; 2) verificação da quantidade de participantes; 3) preenchimento, pelos participantes, dos instrumentos referentes aos dados sociodemográficos, laborais, hábitos de vida e condições de saúde e do *Oldenburg Burnout Inventory* (OLBI). Tal preenchimento foi por meio do aplicativo *Google Forms*®, utilizando-se *link* compartilhado pela equipe de pesquisa a partir da autorização das chefias imediatas. A partir desse *link,* o participante tinha acesso ao TCLE e, caso concordasse com os termos deste estudo, também aos instrumentos de coleta de dados. Foi aplicado um instrumento para identificação de dados sociodemográficos, laborais, hábitos de vida e condições de saúde, elaborado pelos autores, contendo as variáveis sexo, idade, crença religiosa, cor, números de filhos, situação conjugal, escolaridade, renda familiar e individual, tempo de exercício profissional, tempo de experiência na área da segurança pública, carga horária semanal de trabalho, número de vínculos laborais, turno de trabalho, afastamento no último semestre por motivo de saúde, últimas férias, atividades físicas, lazer, tabagismo, alcoolismo, doença crônica, utilização de medicamento e problemas de saúde frequentes. Os autores embasaram-se em artigo publicado em 2022^(14)^ que verificou a associação entre síndrome de *burnout* e violência ocupacional em professores na mesma região deste estudo para a elaboração do instrumento.

Para identificar a presença e os índices do *burnout,* optou-se por um instrumento validado para o Brasil e com bons índices de confiabilidade^(15)^, o OLBI, que avalia as características que podem desencadear o *burnout* e que são associadas às relações e condições de trabalho por meio de uma escala que contém 13 questões e dois fatores, como exaustão, com seis questões, e desligamento do trabalho, com sete. Pode ser aplicado em qualquer contexto ocupacional. Essa escala foi desenvolvida originalmente em 1999 no idioma alemão, sendo traduzida e validada no Brasil em 2018. A licença para utilizar o OLBI neste estudo foi solicitada e concedida pelos autores que a validaram no Brasil.

### Análise estatística

Elaborou-se um banco de dados com dupla digitação, e as análises foram realizadas por meio do *software Statistical Package for the SocialSciences* (SPSS), versão 25, e programa R, versão 3.4.0. Os dados foram analisados por estatística descritiva, mediante o cálculo de frequência e percentual para as variáveis categóricas. Foram categorizados de forma dicotômica, levando-se em consideração o parâmetro médio para configuração das classes. Com vistas a analisar a confiabilidade do OLBI para a presente amostra, realizou-se a análise do alfa de Cronbach, que revelou um índice adequado (α = 0,925).

Posteriormente, foi realizada análise por meio do teste exato de Fisher para verificar a existência de associação das dimensões do *burnout* com as variáveis de caracterização pessoais, ocupacionais, hábitos de vida e condições de saúde. O nível de significância adotado para essas análises foi de α ≤ 0,05.

Visando padronizar a variável caracterizada como desfecho, tendo em vista a distribuição das ocorrências positivas (ter *burnout),* estabelecida em quatro domínios, optou-se por realizar a categorização entre não possuir *burnout* (sem *burnout,* com distanciamento, com esgotamento) e possuir *burnout* (com *burnout*). Por meio dos testes qui-quadrado e exato de Fisher, realizaram-se a análise da razão de chances e as possíveis associações entre as variáveis, caracterizações pessoais, ocupacionais, hábitos de vida e condições de saúde com a síndrome de *burnout.*

Com vistas a estimar um modelo de predição para pertencer à categoria de pessoas que possuíam *burnout,* utilizou-se o Modelo Linear Generalizado de Poisson com variância robusta, obtendo-se, assim, a razão de prevalência. Todos os dados foram considerados significantes com valor de *p* ≤ 0,05.

## RESULTADOS

Participaram deste estudo 131 policiais, sendo 87,0% (n=114) do sexo masculino, 85,4% (n=112) com idade entre 31 e 59 anos, 64,1% (n=84) brancos, 62,5% (n=82) católicos, 63,3% (n=83) com um a dois filhos e 81,6% (n=107) casados ou com companheiros. Em relação à formação, 45,0% (n=59) tinham pós-graduação. Quanto à renda familiar mensal, 47,3% (n=62) manifestaram receber acima de cinco salários mínimos mensais (R$ 6.060,00).

O uso de bebidas alcoólicas foi constatado em 53,4% (n=70); nenhum participante informou que fazia uso de drogas ilícitas; e apenas 8,4% (n=11) eram tabagistas. Em relação aos processos de saúde-doença-cuidado, 93,8% (n=123) não tinham doença crônica; 73,2% (n=96) não utilizavam medicamentos de uso contínuo; 87,0% (n=114) afirmaram não ter problema de saúde frequente; e 76,3% (n=100) praticavam atividades de lazer. Com relação à saúde mental, 65,6% (n=86) apresentaram índices mais elevados de *burnout,* enquanto o restante concentrou-se nas outras respostas (sem *burnout,* com distanciamento e com esgotamento). Somente a variável atividade de lazer associou-se com o *burnout* (*p* = 0,009).

Um pouco mais da metade dos policiais tinha mais de 10 anos de experiência na PMPR (51,9%, n=68), e 57,2% (n=75) possuíam mais de 10 anos de atuação na área da segurança pública (Tabela 1). A maior parte, ou seja, 79,3% (n=104), realizava carga horária semanal acima de 40 horas; 76,3% (n=100) não tinham outro vínculo empregatício; 90,0% (n=118) trabalhavam em turno rotativo; 54,9% (n=72) não precisaram se ausentar do trabalho no período de um ano antes da coleta de dados; e o mesmo número praticava atividade física mais que três vezes na semana. No momento da coleta de dados, 73,2% (n=96) já haviam usufruído de férias nesse ano.

**Tabela 1:** Distribuição da ocorrência de *burnout* segundo as variáveis sociodemográficas, laborais, hábitos de vida e condições de saúde. Paraná, Brasil, 2022 (N= 131)

Burnout		Burnout					
		Sim		Não		OR(IC)	Valor de *p*
		f	%	f	%		
Sexo	Masculino	86	86,9	28	87,5	1,044 (0,418-2,607)	0,926
	Feminino	13	13,1	4	12,5		
Cor da pele	Branca	64	64,6	20	62,5	1,023(0,833-1,257)	0,826
	Não branca	35	35,4	12	37,5		
Crença religiosa	Com crença	95	96,0	30	93,8	1,140(0,642-0,025)	0,603
	Sem crença	4	4,0	2	6,3		
Situação conjugal	Com companheiro	79	79,8	28	87,5	1,570(0,717-1,095)	0,328
	Sem companheiro	20	20,2	4	12,5		
Escolaridade	Ensino superior	80	80,8	24	75,0	1,093(0,837-1,427)	0,480
	Ensino médio	19	19,2	8	25,0		
Renda familiar	Até 5 SM	52	52,5	17	54,8	1,073(0,579-1,991)	0,822
	>5 SM	47	47,5	14	45,2		
Renda individual	< 3 SM	9	9,1	2	6,3		
	3 ou mais SM	90	90,9	30	93,8	1,091(0,811-1,468)	0,614
Anos de exercício profissional	6 -10	50	50,5	13	40,6		
	>10	49	49,5	19	59,4	1,101(0,907-1,338)	0,331
Anos de experiência na segurança pública	Até 10	45	45,5	11	34,4		
	>10	54	54,5	21	65,6	1,116(0,922-1,352)	0,271
Possui outro serviço	Não	72	72,7	28	87,5	2,170(0,825-5,708)	0,087
	Sim	27	27,3	4	12,5		
Turno de trabalho	Rotativo	88	88,9	30	93,8	1,653(0,445-6,135)	0,424
	Fixo	11	11,1	2	6,3		
Dias ausentes no trabalho	Não	53	53,5	19	59,4	1,198(0,647-2,217)	0,564
	Sim	46	46,5	13	40,6		
Bebida alcoólica	Não	44	44,4	17	53,1		
	Sim	55	55,6	15	46,9	1,301(0,711-2,378)	0,392
Tabagismo	Não	91	91,9	29	90,6	1,043(0,716-1,518)	0,818
	Sim	8	8,1	3	9,4		
Doença crônica	Não	92	92,9	31	96,9	2,016(0,314-12,932)	0,418
	Sim	7	7,1	1	3,1		
Uso contínuo de medicamentos	Não	71	71,7	25	78,1	1,302(0,619-2,738)	0,476
	Sim	28	28,3	7	21,9		
Problema de saúde frequente	Não	84	84,8	30	93,8	2,237(0,587-8,522)	0,193
	Sim	15	15,2	2	6,3		

*Teste exato de Fisher; salário mínimo (SM) na data da coleta de dados: R$ 1.212,00 ou US$253,56 (cotação do dólar norte-americano realizada dia 02/06/2022).

Constatou-se a ausência de associação das variáveis ocupacionais com as dimensões do *burnout.* Na Tabela 1, os PMs que não possuíam outro emprego tiveram aproximadamente duas vezes mais chances de não ter *burnout* do que aqueles que possuíam. Não possuir doença crônica demonstrou aproximadamente duas vezes mais chances de não desenvolver *burnout,* e quem não tinha problema de saúde frequente apresentou aproximadamente duas vezes mais chances de não ter *burnout.* A variável atividade de lazer apresentou associação, indicando que quem praticava atividade de lazer tinha 33,6% chances de não desenvolver o *burnout,* quando comparados aos que não a praticavam (OR: 1,336, *p* = 1,008). Esses dados estão sumarizados na Tabela 1.

As variáveis sexo e idade foram mantidas no modelo, por serem consideradas de confusão, contudo não apresentaram contribuição para a sua construção. As variáveis dias ausentes, férias nesse ano, possuir doença crônica e idade apresentaram associação marginal ou limítrofe. A variável turno rotativo mostrou-se fator protetivo para ocorrência de *burnout,* uma vez que apresentou coeficiente negativo (β= -0,363, *p* = 0,019) (Tabela 2).

**Tabela 2:** Análise dos fatores individuais na predição da variável desfecho *burnout* (sim ou não). Paraná, Brasil, 2022 (N= 131)

Parâmetro	^ß^	Valor de *p*	RP	IC (95%)	
				Inferior	Superior
(Intercepto)	1,372	<0,001	3,944	2,162	7,194
Sexo					
Masculino	0,024	0,755	1,024	0,883	1,187
Feminino	Ref.				
Turno de trabalho					
Rotativo	−0,363	0,019	0,695	0,513	0,943
Fixo	Ref.				
Dias ausentes					
< 15 dias	−0,101	0,083	0,904	0,807	1,013
>15 dias	Ref.				
Férias esse ano					
Não	0,078	0,083	1,082	0,990	1,182
Sim	Ref.				
Atividade física					
Não	0,100	0,044	1,106	1,003	1,219
Sim	Ref.				
Possui doença crônica					
Não	−0,166	0,058	0,847	0,713	1,006
Sim	Ref.				
Idade (anos)	−0,008	0,066	0,992	0,983	1,001
Realiza atividades de lazer					
Não	0,077	0,049	1,080	1,000	1,165
Sim	Ref.				
Situação conjugal					
Com companheiro	−0,121	0,025	0,886	0,797	0,985
Sem companheiro	Ref.				

*Regressão de Poisson com variância robusta; Ref. – referência; β=coeficiente de regressão; RP – Razão de Prevalência; IC – Intervalo de Confiança.

Não praticar exercício físico contribuiu positivamente para a ocorrência de *burnout* (β= -0,100, *p* = 0,044), assim como não realizar atividades de lazer também contribuiu positivamente para sua ocorrência (β= 0,077, *p* = 0,049). A situação conjugal com companheiro foi protetiva para o desenvolvimento de *burnout* (β= -0,121, *p* = 0,025) (Tabela 2).

A Tabela 2, a seguir, apresenta a análise de regressão. Podem-se notar no modelo variáveis que demonstraram vulnerabilidade para o desenvolvimento do *burnout,* como turno de trabalho fixo, possuir doença crônica, não realizar atividade de lazer e prática de atividades físicas e não ter companheiro.

## DISCUSSÃO

A maioria dos participantes deste estudo (87,0%) demonstrou índices mais elevados de *burnout,* achado esse que é reportado na literatura científica^(16)^, sobretudo considerando os diversos marcadores associados ao cotidiano dessa profissão, como a questão do sigilo e a atuação diante de estressores nem sempre controláveis.

As mulheres que exercem a função de PMs tendem a apresentar índices mais elevados de estresse, quando comparadas aos homens dessa profissão^(17)^, o que pode se dar em função dos demais papéis sociais ocupados em função do gênero em nosso país. Ressalta-se que, no presente estudo, observou-se número maior de participantes do sexo masculino (85,4%), o que já era esperado, pois a categoria profissional militar é composta majoritariamente por homens. Estudo realizado em 2019 no estado do Rio Grande do Sul também observou tal prevalência^(18)^.

Embora a literatura científica possa sugerir associações entre idade, cor de pele e *burnout* ^(19)^, o presente estudo mostrou associação apenas marginal/limítrofe entre essas variáveis. Em relação à religiosidade/espiritualidade, a maioria da amostra identificou-se como sendo católica (62,5%). A religiosidade/espiritualidade pode ser entendida como um recurso empregado no âmbito individual e no coletivo, o qual busca enfrentar os eventos adversos da vida, que podem ter repercussões emocionais e sociais. Assim, acredita-se que a crença religiosa pode ser um recurso para os participantes que possuem alguma crença, já que a religiosidade pode ressignificar o sentido da existência humana e ampliar conceitos e consciência. A busca do PM por meio da religiosidade e do desenvolvimento da espiritualidade pode auxiliar no espaço de construção e reflexão quanto ao momento de vida atual e futuro de cada um^(20)^.

Verificou-se que 63,3% dos PMs possuíam de um a dois filhos, dados próximos aos resultados apresentados por estudo que objetivou avaliar a percepção dos índices de atividade física e dos indicadores de *burnout* em PMs na cidade de Belo Horizonte no estado de Minas Gerais (MG), sendo que 61,0% dos participantes possuíam filhos^(21)^. A realização das atividades físicas é um fator protetivo contra o *burnout,* achado esse enfatizado no presente estudo. Em relação à parentalidade, há consenso na literatura de que ser pai ou mãe constitui uma possível causa de proteção ou menor índice de doenças como o *burnout* ^(22)^. No entanto, essa associação não se mostrou significativa no presente estudo. Aventa-se, aqui, que a relação protetiva possa ocorrer quando a parentalidade se mostra positiva e quando as relações entre pais e filhos é saudável, com menor índice de conflitos. Para suportar tal hipótese, a variável parentalidade deveria ter sido explorada de modo mais consistente na presente investigação.

Com relação ao nível de escolaridade, embora, para ser admitido na Polícia Militar, seja solicitada apenas conclusão do nível médio, verificou-se, na presente investigação, que parte dos policiais tinha se aprimorado, ou seja, 45% deles tinham pós-graduação. Ressalta-se que existe oferta de oportunidade de graduação/pós-graduação presencial e a distância em várias instituições de ensino superior na cidade onde o estudo foi desenvolvido, bem como facilidade de locomoção entre o trabalho e as instituições de ensino na cidade onde os policiais residem, desconto na mensalidade por ser PM e ofertas de bolsas para esse público. Esse cenário pode ter contribuído para a ampliação do nível de escolaridade nessa amostra. Contudo, estudo realizado em Belo Horizonte, MG, com 195 PMs, mostrou que a maioria deles possuía somente o ensino médio^(21)^. Pesquisa realizada com professores com *burnout* demonstrou que, quanto maior o nível de escolaridade, maior probabilidade de desenvolvê-lo^(14)^.

Em relação à renda individual e familiar mensal, nesta pesquisa, verificou-se que a população investigada recebia acima de três salários mínimos, baseados no valor de R$ 1.212,00 (ano de 2022), ou seja, quantidade inferior à classe média brasileira^(23)^. Pesquisa realizada no Rio Grande do Sul mostrou que houve insatisfação laboral dos PMs em relação ao salário e às promoções na carreira^(24)^. A faixa salarial pode influenciar o risco de desenvolver o *burnout.* Salários menores podem levar a vivenciar a tensão desencadeada por uma renda insuficiente para suas necessidades. Já a renda maior, provavelmente, é fruto de mais de um vínculo empregatício, fato que pode levar ao desgaste físico e/ou mental^(25)^, pelo acúmulo de funções e jornadas mais rígidas de trabalho.

Diferente do estudo realizado na cidade de Belo Horizonte, MG, cujos PMs tiveram baixo adesão à atividade física^(21)^, os resultados deste estudo mostraram que a maioria praticava atividade física mais de três vezes por semana. Em concordância com esses resultados, não praticar exercício físico pode contribuir positivamente para a ocorrência de *burnout;* portanto, quanto maior for o nível de atividade física, menores as chances de desenvolver *burnout* ^(26)^.

A maioria dos participantes fazia uso de bebida alcoólica (53,4%); nenhum informou que utilizava drogas ilícitas; e a minoria era tabagista. O abuso de substâncias representado pela dependência do álcool é associado comumente aos transtornos depressivos e ao *burnout* ^(27)^. As atividades de policiamento podem causar distúrbios do sono e, consequentemente, erros administrativos, condução perigosa de veículos, sentimentos de raiva de maneira descontrolada, violação da segurança por conta do absenteísmo e fadiga e exposição constante à tragédia humana, levando ao risco dos PMs desenvolverem transtornos mentais, com potencial para o abuso de álcool e outras drogas^(28)^.

A realidade dessa atividade, inúmeras vezes, é permeada por tensão, pressão, cobrança, sofrimento psíquico, insatisfação com as condições de trabalho, falta de preparo para a função e obrigação “incondicional” de não demonstrar fragilidade, fatores esses relacionados ao surgimento de transtornos psicológicos^(29)^, o que pode também aumentar as chances de o profissional desenvolver sintomas de *burnout,* como constatado neste estudo.

Nesta pesquisa, 93,8% dos policiais não apresentavam doença crônica, um fator protetor para *burnout,* e 73,2% não utilizavam medicamentos de uso contínuo. Além disso, 87,0% afirmaram não ter problema de saúde frequente, contudo observou-se um grande número de PMs com *burnout.* Observou-se que não possuir doença crônica faz com que a pessoa tenha aproximadamente duas vezes mais chances de não desenvolver *burnout.* Estudos demonstraram que os fatores relacionados ao adoecimento dos PMs foram não conseguir prestar socorro em tempo hábil, ser impotente diante de óbitos de pessoas que não conseguiram auxiliar, interpretações negativas veiculadas ao PM, falta de lazer, baixa qualidade de vida e vitimização. Tais situações podem interferir na qualidade de vida desses trabalhadores, ocasionando problemas de saúde e uso de medicações^(30, 31, 32, 33)^.

O trabalho do PM pode causar seu afastamento da sociedade, dos familiares, dos amigos e das relações interpessoais^(34)^ e, muitas vezes, as condições nas quais trabalha são precárias^(4)^. O fato de não realizarem atividade de lazer contribui significativamente para a ocorrência do *burnout,* e observou-se, neste estudo, que a prática de atividades de lazer os leva a 33,6% mais chances de não desenvolver o *burnout,* quando comparados aos que não praticavam tais atividades.

Pesquisa que objetivou investigar os índices de estresse ocupacional e engajamento no trabalho em PMs do Paraná também revelou que a maioria dos participantes era casada ou tinha companheiro^(4)^. Em relação ao estado civil, algumas pesquisas atestam que a conjugalidade é uma condição que implica menor disposição aos sintomas de *burnout,* dado esse corroborado pelo presente estudo^(22)^. Assim, ter um companheiro foi um fator protetivo contra a ocorrência do *burnout,* o que pode ser explicado tanto pelo fato desse parceiro oferecer apoio emocional quanto pelo fato desses relacionamentos interpessoais oferecerem satisfação e sensação de bem-estar. Em uma profissão fortemente marcada pelo estresse e pela necessidade de sigilo, o compartilhamento do cotidiano com um parceiro pode ser um fator que predisponha o profissional a uma maior abertura e a um maior cuidado em relação à sua saúde mental.

Este estudo evidenciou também que, nos PMs que tinham mais de 10 anos de experiência e mais de 10 anos de atuação na área da segurança pública, com carga horária semanal maior que 40 horas e com expressiva experiência e tempo de atuação, o *burnout* fez-se presente. Pesquisa realizada nos Estados Unidos demonstrou que, quanto mais tempo de serviço, maior o estresse no ambiente de trabalho^(16)^, além de maior sentimento de ansiedade e sofrimento^(35)^.

Estudo de revisão apontou que trabalhadores mais experientes sentem-se seguros, têm controle sobre o seu ambiente laboral e lidam melhor com as demandas de trabalho, estando mais protegidos contra o *burnout* ^(36)^. Todavia, na presente pesquisa, os achados discordaram desse resultado reportado na literatura científica, haja vista que não houve associação entre a idade e o tempo de experiência com a variável *burnout.*

Vale mencionar que os PMs que não possuíam outro emprego tinham aproximadamente duas vezes mais chances de não apresentarem *burnout* do que os que possuíam. Portanto, ter um vínculo apenas de trabalho mostrou-se um fator protetivo para o *burnout* ^(22)^. Destaca-se que 76,3% dos pesquisados tinham apenas um vínculo de trabalho. Contudo, há menções na literatura que baixos salários fazem com que os PMs busquem aumentar a renda com outros trabalhos^(37)^, o que pode favorecer a presença do *burnout.*

Neste estudo, 90,0% dos PMs trabalhavam em turno rotativo, e esse fator foi protetivo contra a ocorrência de *burnout.* Fatores como ausência de folgas, trabalho em turnos, mudança de escala sem aviso prévio, lidar com hierarquia e burocracia rígidas, atender crianças maltratadas, ter que tirar a vida de alguém se necessário, ver colegas de trabalho serem mortos durante o serviço, comparecer aos tribunais em suas folgas, falta de apoio do supervisor, dimensionamento de pessoal ineficiente, trabalhar nos fins de semana e demanda de trabalho estão relacionados ao surgimento de sintomas psíquicos^(30, 31, 32)^.

Nesta pesquisa, 54,9% dos pesquisados não se ausentaram do trabalho no ano de coleta de dados, e 73,2% já haviam tirado férias nesse mesmo ano. Tais variáveis e sua razão de prevalência na população mostraram associação marginal/limítrofe com o *burnout.* Ajustar dias de folga, não deixar acumular férias, adequar salários e proporcionar um plano de moradia e saúde para os PMs podem ser ações que evitem prejuízos à saúde desses profissionais^(38)^.

Diante dos resultados apresentados até então, é possível inferir que as atividades policiais necessitam de atenção tanto por parte dos gestores quanto das políticas públicas, tendo como objetivo o manejo dos fatores estressantes e consequente controle do *burnout* nessa população. O trabalho dos PMs é de grande importância para o país, e sua natureza vai além do condicionamento físico, exigindo bom desempenho emocional^(17)^.

Nesse contexto, torna-se importante mencionar que políticas públicas em níveis federal, estadual e municipal para o enfrentamento desse fenômeno são primordiais. Sugere-se a utilização de estratégias que promovam redução da sobrecarga de trabalho, bom relacionamento interpessoal entre os níveis hierárquicos e melhoria das condições laborais e da organização do trabalho para diminuir os fatores associados ao *burnout.* Sobretudo, evidencia-se a importância de ter o olhar atento aos riscos laborais a que esses trabalhadores estão expostos, investindo e disponibilizando recursos para que haja mais profissionais de saúde de forma interdisciplinar contribuindo para a promoção da saúde desses trabalhadores responsáveis pela segurança pública.

### Limitações do estudo

Como limitações do estudo, relata-se que esta pesquisa tem delineamento transversal e não permite estabelecer relações entre causa e efeito. Importante destacar, ainda, a dificuldade de encontrar investigações sobre a temática em questão, o que pode limitar a discussão dos achados e o cotejamento dos mesmos diante da literatura científica.

### Contribuições para as áreas da saúde, enfermagem, ou políticas públicas

Pressupõe-se que o presente estudo possa subsidiar a compreensão dos fatores que levam os PMs a ter *burnout.* Apresenta-se como uma possibilidade de novos estudos, principalmente nas áreas da saúde do trabalhador, saúde mental e segurança pública, ao propiciar que se repense a saúde dos PMs com uma visão integral. Esses dados podem, em conjunto, promover avanços na produção de conhecimentos científicos e práticos a respeito dessa temática, possibilitando a elaboração de medidas e políticas voltadas para essa população. Há necessidade de projetos que visem aumentar a qualidade de vida no trabalho desses profissionais e os incentivos de pesquisas científicas, a fim de avaliar resultados e alcances de sua prática em diversas regiões.

Contribui sobremaneira para que esses profissionais não sejam silenciados em relação ao seu sofrimento psíquico, que é um dos compromissos reforçados a partir desta pesquisa e dos dados aqui compartilhados. São prementes as estratégias e políticas públicas direcionadas à prevenção do *burnout,* bem como ações intervencionistas que reduzam o *burnout* e visem à manutenção da saúde física e mental dos PMs como: o monitoramento do ambiente de trabalho; a oferta de práticas integrativas e complementares; o acompanhamento desses policiais por profissionais de saúde de forma interdisciplinar; a promoção de redes de apoio; e a discussão de tal temática com os gestores e a sociedade em geral, enfatizando a necessidade que esses profissionais têm de serem ouvidos e cuidados.

## CONCLUSÕES

Observaram-se, neste estudo, índices elevados de *burnout* nos policiais investigados. Resultados importantes foram evidenciados, como que não ter outro emprego e não ter doença crônica leva a uma maior chance de não desenvolver o *burnout,* assim como que quem não apresentou problemas de saúde frequentes.

A variável turno rotativo mostrou-se um fator protetor contra a ocorrência de *burnout,* assim como a situação conjugal (ter companheiro), reforçando a importância tanto de fatores laborais (turno) quanto de relacionamentos interpessoais satisfatórios (conjugalidade) para a manutenção da saúde emocional e da qualidade de vida, o que pode refletir-se em menores índices de *burnout* quando esses fatores protetivos estão presentes.

De modo análogo, mas em sentido oposto, não praticar exercício físico e não realizar atividades de lazer contribui para a ocorrência de *burnout.* Diante desses achados, é fundamental que ocorram discussões sobre a saúde mental no trabalho desses profissionais, integrando tanto elementos laborais passíveis de ajustes, como os turnos de trabalho, quanto o fomento ao investimento em ações que extrapolam o contexto de trabalho, como incentivo à prática de exercícios físicos, atividades de lazer e fortalecimento de relacionamentos interpessoais, entre os quais situamos, a partir deste estudo, a conjugalidade.
